# Evaluation of the Effect of *Dracocephalum moldavica* Mucilage and Nanoemulsion of *Satureja hortensis* Essential Oil Edible Coating on Microbial, Chemical, and Organoleptic Properties of Rainbow Trout Fish Fillet at Refrigerator Temperature

**DOI:** 10.1002/fsn3.70445

**Published:** 2025-07-26

**Authors:** Kosar Maghsoudi, Razieh Partovi, Shohreh Alian Samakkhah

**Affiliations:** ^1^ Department of Food Hygiene Faculty of Veterinary Medicine, Amol University of Special Modern Technologies Amol Iran

**Keywords:** biodegradable covering, meat, nanoemulsion, plant derived polysaccharides, savory, shelf life

## Abstract

This study evaluated the use of an edible coating made from *Dracocephalum moldavica* seed mucilage with *Satureja hortensis* essential oil emulsion and nanoemulsion to extend the shelf life as well as preserve the sensory qualities of rainbow trout fillets. Fillets were split into four groups: uncoated (CON), 1% *D. moldavica* mucilage coating (M), mucilage coating containing 2% *S. hortensis* EO (M + EO), and mucilage coating containing 2% nanoemulsion of *S. hortensis* EO (M + NEO). After coating, the fish fillets were assessed for microbial (Total Viable Count, psychrotroph, coliform, *Staphylococcus aureus* , Lactic Acid Bacteria, and yeast and mold), chemical (pH, Total Volatile Basic Nitrogen, and Thiobarbituric Acid Reactive Substances) and organoleptic (taste, color, texture, odor, and overall acceptability) properties at specified intervals (0,2,4,7 days). Carvacrol (40.23%), p‐cymene (24.42%), and γ‐terpinene (21.76%) were the principal compounds of *S. hortensis* EO. The nanoemulsion showed an average particle diameter of 135.6 ± 3.81 nm and a zeta potential of −51.26 ± 1.70 mV. Throughout the study, no significant difference was observed between the CON and M groups, but significant differences were found between these two groups and the M + EO as well as M + NEO groups (*p* < 0.05), leading to an extension of shelf life by more than 3 days. The findings of the chemical tests revealed a similar trend to the microbial results, indicating a significant difference between the CON as well as M group and the groups containing the emulsion and nanoemulsion of *S. hortensis* EO. Sensory scores diminished over time but were best maintained in the nanoemulsion‐coated samples. According to the results of the present study, the edible coating of *D. moldavica* seed mucilage containing the emulsion and nanoemulsion of *S. hortensis* EO can be employed to enhance the shelf life of rainbow trout fillets.

## Graphical abstract

The results of the present study demonstrated that the edible coating containing *D. moldavica* mucilage and *S. hortensis* EO emulsion and nanoemulsion could improve the microbial and chemical properties of rainbow trout fillets, extending their shelf life by more than 3 days. However, throughout the study, mucilage‐only group did not show any significant differences compared to the control. Sensory scores decreased over time but were best maintained in the nanoemulsion‐coated rainbow trout samples.

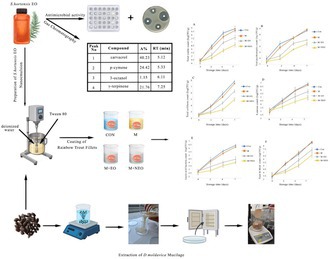

## 1 Introduction

Today, there is growing demand for healthy, accessible, adaptable, affordable, and high‐quality food (Gokoglu 2020). Fish meat is recognized as an important food source for humans and contains significant amounts of protein, vitamins, and omega‐3 fatty acids. Thus, the demand for fish products in domestic and international markets has grown (Patil et al. 2020). Nevertheless, fish meat is susceptible to microbial and chemical spoilage owing to its high pH and water activity, as well as its high content of unsaturated fatty acids plus free amino acids (Zhelyazkov and Stratev 2019). Fish spoilage is a result of biological changes, such as lipid oxidation, protein degradation, enzymatic reactions, and metabolic activity of microorganisms, leading to the generation of off‐odors, off‐flavors, and deterioration of quality during storage (Aref et al. 2022). Since rainbow trout contains 65%–80% moisture, a high fat content, free amino acids, and 9%–18% various non‐protein nitrogenous compounds, it experiences numerous biochemical, physical, and microbial changes. Safety, prevention of spoilage, and the maintenance of high‐quality fish during storage are primary concerns for both consumers and producers. In light of these issues, adoption of innovative and complementary technologies for food protection has attracted significant interest. This involves food packaging with antimicrobial properties, such as biodegradable films and edible coatings incorporated with antimicrobial composites and natural antioxidants (Azizi et al. 2024).

Environmental concerns regarding the negative influence of plastic packaging residues have prompted food industry experts to develop a new type of films and edible coatings to preserve food quality (Heydari et al. 2020). The usage of edible coatings can help overcome some of these issues while providing additional benefits such as improved health through nutrient enrichment while also acting as carriers for natural preservatives such as antioxidants and antimicrobial agents (Andriani and Handayani 2023). Edible coatings can be created using a range of natural substances sourced from biodegradable agricultural materials or byproducts of food processing including polysaccharides, proteins, and lipids (Saffari Samani et al. 2023). Among various polysaccharides, plant‐derived mucilage is widely utilized in different food industries thanks to its extensive and valuable applications. Plant mucilage is extensively applied as an active ingredient for formulating pharmaceutical, functional, and food products owing to its health properties (anticancer and immune‐stimulating) and nutritional benefits (Tosif et al. 2021). Further, as a hydrocolloid, it possesses various properties and is employed in the food industry as a thickening agent, texture enhancer, stabilizer, and natural gelling agent (Kassem et al. 2021).

*D. moldavica* is an annual herbaceous plant of the Lamiaceae family, an essential oil plant as well as an oilseed crop (Acimovic et al. 2019). One of the unique features of *D. moldavica* seeds is their excellent mucilage content. The mucilage from this plant has great biological potential as a new antioxidant edible film with favorable characteristics that can be used for packaging food products (Beigomi et al. 2018). Unfortunately, there is relatively limited information on *D. moldavica* seed mucilage, its chemical composition and its food applications and no edible coating has been prepared using this mucilage so far. The main studies regarding this plant have focused mostly on the EO and properties of the green parts of the plant. However, its seed mucilage can be applied as a raw material for nutritional supplements, food supplements, and applications in the food industry.

EOs are highly hydrophobic compounds generated by plant organs (Bassolé and Juliani 2012). They are utilized in various industries, including food, pharmaceutical, medical, cosmetic, and hygiene (Baser and Buchbauer 2009). Some of the most important functions of EOs are inhibiting the growth of pathogens, delaying food spoilage and oxidation, and enhancing organoleptic quality. The use of natural products like EOs as antibacterial compounds can mitigate health risks and economic losses caused by microorganisms in food (Ehsani et al. 2017).

*S. hortensis* , a member of mint family, is used as a medicinal plant, spice, and fresh herb. Based on studies, thymol, carvacrol, α‐pinene, β‐ pinene, and p‐cymene are among the principal compounds of *S. hortensis* EO (Chambre et al. 2020). These compounds exhibit various capabilities in treating serious diseases such as diarrhea, cardiovascular diseases, cancer, and Alzheimer's. They can also serve as antioxidants, anti‐inflammatory agents, antimicrobials, antifungals, and drug resistance inhibitors (Góra et al. 1996; Novak et al. 2006; Oke et al. 2009). The low solubility of EOs in water, their high vapor pressure, as well as their physical and chemical instabilities have restricted their usage. Further, the unpleasant smell and taste of EOs in food products have led to consumer dissatisfaction. Thus, there is a growing focus on methods to mitigate the negative effects of EOs (Rastegae 2019). The application of nanotechnology to disperse plant EOs in water has enhanced the solubility and stability of these compounds (Firoozi et al. 2020). Among nanoemulsions, oil‐in‐water emulsions with small particle sizes ranging from 100 to 500 nm have attracted more attention thanks to their safety and compatibility with water. Nanoemulsions also possess unique properties, such as high optical clarity and enhanced physical stability, making them beneficial for food applications compared to conventional emulsions (Farshbaf‐Sadigh et al. 2021; Jafarinia et al. 2022).

Previously, studies have been performed on the effects of chia mucilage coating with sage EO on rainbow trout (Çoban et al. 2016), fish gelatin enriched with oregano EO on rainbow trout fillet (Hosseini et al. 2016), tomato seed mucilage isolate—whey protein enriched with shallot EO (Izadi et al. 2024), and nanoemulsion based on herb EOs (rosemary, laurel, thyme and sage) on rainbow trout (Ozogul et al. 2017). Studies have also been undertaken on edible coatings of plant seed mucilage on meat, including cinnamon EO as well as its application with *Malva sylvestris* seed mucilage on lamb along storage (Sabahi et al. 2022), nanoemulsion of citrus EO and *Lallemantia iberica* seed mucilage on lamb (Alizadeh Behbahani and Imani Fooladi 2018), *Ocimum basilicum* seed mucilage with *Mentha pulegium* EO to enhance the quality and shelf life of beef (Tanavar et al. 2021), and *Plantago major* seed mucilage with *Anethum graveolens* EO on beef in the refrigerator (Behbahani et al. 2017).

In spite of the undeniable advantages of the mucilage from *D. moldavica* seeds, no studies have been conducted to apply this mucilage for creating food edible coatings. No study has been carried out on the properties of *S. hortensis* EO nanoemulsion as well as its application as part of an edible coating to extend the shelf life of food products. Thus, this study aimed to examine the effects of edible coatings based on *D. moldavica* seed mucilage combined with *S. hortensis* EO emulsions and nanoemulsions on the microbial, chemical, and sensory properties of rainbow trout fillets along refrigeration.

## 2 Materials and Methods

All chemicals and solvents including Dimethyl Sulfoxide (DMSO), Tween 80, 1‐butanol, and tellurite egg yolk were obtained from Sigma‐Aldrich (USA). Culture media such as nutrient broth, nutrient agar, peptone water, Plate Count Agar (PCA), MRS, Violet Red Bile Agar (VRBA), Baird Parker Agar (BPA) and Potato Dextrose Agar (PDA) were obtained from Merck‐Millipore (Darmstadt, Germany).

### 2.1 Preparation and Analysis of *S. hortensis* EO

*S. hortensis* EO prepared from Barij EO Company (Kashan, Iran) was analyzed via GC–MS (Thermoquest 2000, Manchester, UK) using a capillary column. The conditions were as follows: an initial temperature of 50°C, a 2.5°C/min program rate, a final temperature of 265°C, and an injector temperature of 250°C. Helium served as the carrier gas (1:120), with mass spectrometry in electron ionization mode at 70 Ev (Partovi et al. 2019).

### 2.2 Determination of Minimum Inhibitory Concentration (MIC) and Minimum Bactericidal Concentration (MBC)

To determine MIC and MBC of *S. hortensis* EO against *Staphylococcus aureus* , *Escherichia coli* , *Salmonella typhimurium* , and *Listeria monocytogenes* , a 0.5 McFarland standard of the studied bacteria was prepared and diluted to 10^6^ CFU/mL. Different concentrations of the EO (0.312–160 mg/mL) were then prepared using DMSO. For MIC determination, nutrient broth, EO, and bacteria (200 μL) were placed in the wells of a microplate and incubated. The lowest concentration of EO that presented no turbidity or microbial growth was considered as MIC. To specify the MBC, the wells without turbidity were inoculated onto nutrient agar and then incubated. The lowest concentration of EO showing no bacterial growth was considered as MBC (Panahi and Mohsenzadeh 2022).

### 2.3 Evaluation of Antimicrobial Activity of *S. hortensis* EO by Disk Diffusion Method

The antibacterial activity of *S. hortensis* EO against foodborne pathogens ( *S. aureus* , *E. coli* , *S. typhimurium* , and *L. monocytogenes* ) was ascertained using the disk diffusion method. Varying concentrations (10–320 mg/mL) of the EO were tested. Bacterial suspensions were standardized to 0.5 McFarland and inoculated on nutrient agar plates. Next, sterile paper disks were impregnated with DMSO and the EO, and then placed on the plates. Disks containing DMSO served as a negative control, while commercial disks containing chloramphenicol and streptomycin were regarded as positive controls. Plates were incubated at 37°C for 24 h, with inhibition zone diameters measured in millimeters (Santos et al. 2019).

### 2.4 Extraction of *D. moldavica* Mucilage

Mucilage was extracted according to Beigomi et al. (2018) and Noshad et al. (2021) with some modifications. Cleaned seeds of *D. moldavica* were macerated in distilled water (20:1) for 1 h at 45°C while stirring at 500 rpm. The mixture was filtered, oven‐dried at 45°C, and left still in a cool, dry place. Mucilage yield was calculated as the dry mucilage weight relative to seed weight (Koocheki et al. 2010; Noshad et al. 2021).

### 2.5 Preparation of *S. hortensis* EO Nanoemulsion

The *S. hortensis* EO nanoemulsion was prepared based on a modified method by Zanganeh et al. (2023). Non‐ionic surfactant Tween 80, *S. hortensis* EO, and distilled water were used for the preparation of the base emulsion with a ratio of 3:2:95 weight/weight and homogenization by an Ultra‐Turrax at 10,000 g for 2 min. The nanoemulsion was obtained after sonication treatment of the emulsion at 20 kHz frequency, 200 W input power, and 30% amplitude by an Ultrasonicator (UP400S model, Hielscher Co., Germany) for 15 min.

### 2.6 Analysis of Particle Size and Zeta Potential of *S. hortensis* EO Nanoemulsion

Particle size and distribution were analyzed through dynamic light scattering. The zeta potential of the nanoemulsion was evaluated using a zeta sizer (SZ‐100 Nanopartica Series Instruments, Horiba, Japan) (Liu and Liu 2020; Ebrahimian and Mohsenzadeh 2023).

### 2.7 Preparation of Treatments

The edible coating was prepared following Alizadeh Behbahani et al. (2021) and Heydari et al. (2020) with slight modifications. The key components included *D. moldavica* seed mucilage and *S. hortensis* EO. 1 g of the *D. moldavica mucilage* was mixed with distilled water to make up to 100 mL and heated and agitated using a magnetic stirrer. 2% v/v *S. hortensis EO* (in the form of emulsion and nanoemulsion) was added to the mixture. Subsequently, four groups were created: control (CON), *D. moldavica* mucilage (M), mucilage with *S. hortensis* EO (M + EO), and *D. moldavica* mucilage with *S. hortensis* EO nanoemulsion (M + NEO).

### 2.8 Coating of Rainbow Trout Fillets

Fish fillets obtained from a reputable Amol rainbow trout farm were skinned, transported on ice, washed, and drained. Slices were coated for 1 min, dried under a microbiological hood for 10 min, packaged in polyethylene bags, and finally stored at 4°C (Sabahi et al. 2022). The control group was immersed in distilled water.

### 2.9 Microbiological Analysis

The microbial load of fish fillets along cold storage was analyzed for Total Viable Count (TVC), psychrotrophs, coliforms, *S. aureus* , Lactic Acid Bacteria (LAB), as well as yeast and mold. Then, 10 g of fillet was aseptically transferred to stomacher bags with 90 mL of 0.1% peptone water for serial dilutions. TVC was counted on PCA at 37°C for 48 h, psychrotrophs at 7°C for 10 days, coliforms on VRBA at 37°C for 24 h, *S. aureus* on BPA at 37°C for 48 h, LAB on MRS Agar at 30°C for 48 h, together with yeast and mold on PDA at 25°C for 5 days. Results were expressed as log_10_ CFU/g (Hosseinzadeh et al. 2020; Panahi and Mohsenzadeh 2022; Jooyandeh et al. 2023).

### 2.10 Chemical Analysis

#### 2.10.1 pH

Approximately 10 g of each fish fillet sample was added to 90 mL of sterilized distilled water, mixed, and then homogenized for 2 min. The pH of each sample was measured via digital pH meter electrodes (Mettler Toledo, USA) at room temperature (Shahrampour and Razavi 2023).

#### 2.10.2 Total Volatile Basic Nitrogen (TVB‐N)

The content of TVB‐N in the fish fillet samples was determined based on the method proposed by AOAC (1995). Here, 10 g of the samples was used, with the results expressed as milligrams of nitrogen per 100 g of fish fillet (Hosseinzadeh et al. 2020).

#### 2.10.3 Thiobarbituric Acid Reactive Substances (TBARS)

Specifiaclly, 200 mg of homogenized fish fillet was mixed with 25 mL of 1‐butanol. TBARS reagent (200 mg TBARS in 100 mL 1‐butanol) was prepared, filtered, and refrigerated. Next, 5 mL of the previous sample mixture was added to a clean, dry test tube containing 5 mL of the TBARS reagent. All tubes were placed in a water bath at 95°C for 1 h. Once the tubes were cooled to room temperature, absorbance was measured at 530 nm using a spectrophotometer (Hanon, China). A control tube contained only the TBARS reagent. TBARS content (mg/kg) was calculated accordingly (Shahrampour and Razavi 2023):

$$\text{TBARS}=50\times \left[\left(\text{Absorbance of the sample}-\text{Absorbance of the control}\right)/200\right]$$

### 2.11 Sensory Analysis

To evaluate the sensory characteristics of the fish fillet samples, including color, odor, taste, texture, appearance, and overall acceptability, 10 g of fish fillet from each group was fried in oil (Kilincceker et al. 2009). All samples were coded with random three‐digit numbers, with a 9‐point acceptability scale (1 = lowest and 9 = highest) employed. Samples receiving scores above 4 were considered acceptable. For the evaluation, a panel of 10 trained individuals from the staff and students of Amol University of Special Modern Technologies was employed (Saffari Samani et al. 2023). Sensory evaluation was carried out in a room with a mix of natural and artificial light, room temperature and dimensions of 3 × 4 m.

### 2.12 Statistical Analysis

To compare microbial and chemical variations over the study period in every test group, a linear mixed model for repeated measures and Bonferroni post hoc test were utilized. For between‐group comparisons at different times, one‐way ANOVA and Tukey post hoc test were employed. The Friedman non‐parametric test was applied to ascertain changes in sensory variables over the study period within each group. Further, the Kruskal‐Wallis non‐parametric test was utilized to compare groups at each time point. The results were reported based on mean and standard deviation. Data analysis was carried out using SPSS version 25. In all analyses, a significance level of less than 5% was considered.

## 3 Results and Discussion

### 3.1 Yield of *D. moldavica* Mucilage

In this study, the yield of mucilage extraction from *D. moldavica* seeds was calculated as 10%, which is similar to the yield reported by Beigomi et al. (2018), who found a yield of 11.3% for *D. moldavica* seeds mucilage. The chemical composition of *D. moldavica* seeds mucilage revealed 4.28% protein, 7.16% moisture, 15.50% ash, 8% fat, and 65.04% carbohydrate. While numerous studies have been performed on the EO and chemical composition of *D. moldavica* , there is relatively scant information on the *D. moldavica* seed mucilage, with the only comprehensive study in this area conducted by Beigomi et al. (2018). This demonstrates a need for further research on the applications as well as properties of *D. moldavica* mucilage.

### 3.2 Chemical Composition of *S. hortensis* EO

The findings of the analysis of the chemical compounds in *S. hortensis* EO using GC/MS are reported in Table 1. In this study, 18 chemical compounds were identified, accounting for 97.04% of the EO. The results revealed that the highest amounts of compounds in *S. hortensis* EO were carvacrol (23.40%), p‐cymene (24.42%), and γ‐Terpinene (21.76%). These compounds were also verified by other studies, but with varying percentages which may be discrepancies due to differences in weather conditions, geographic regions, plant species, and soil types (Ejaz et al. 2023). In a similar study by Yazdanpanah Goharrizi (2015), the highest compounds in summer *S. hortensis* EO were also carvacrol (23.41%) and γ‐Terpinene (36.49%). Overall, in line with the results of the present study, many studies have also shown that the major compounds of the savory genus are phenolic monoterpenes such as carvacrol and thymol, which are often accompanied by γ‐terpinene, p‐cymene, and linalool. These phenolic compounds possess antioxidant and antimicrobial properties (Novak et al. 2006).

**TABLE 1:** Chemical composition of *S. hortensis* EO.

Peak No	Compound	A%	RT (min)
1	Carvacrol	40.23	5.12
2	p‐Cymene	24.42	5.33
3	3‐Оctanol	1.15	6.11
4	γ‐Terpinene	21.76	7.25
5	1‐octen‐3‐ol	0.36	8.19
7	Linalool	0.12	9.43
8	cis‐Sabinenhydrate	0.32	11.14
9	Terpinen‐4‐ol	0.64	12.35
10	α‐Terpineol	0.07	12.51
11	Neral	0.11	14.22
12	Thymol	0.65	15.34
13	α‐Pinene	1.02	17.36
14	α‐Myrcene	0.17	20.35
15	β‐Caryophyllene	2.33	21.05
16	β‐Bisabolen	2.18	23.15
17	Spatulenol	0.24	24.44
18	3‐Thujene	1.12	25.17
19	α‐Myrcene	0.15	26.21
Total		97.04	

### 3.3 Antibacterial Activity of *S. hortensis* EO by Microbroth Dilution and Disk Diffusion Methods

The antibacterial activity of *S. hortensis* EO was assessed using microbroth dilution and disk diffusion methods, with results presented in Tables 2 and 3. The MIC and MBC of the *S. hortensis* EO against *S. aureus* , *E. coli* , *S. typhimurium* , and *L. monocytogenes* were determined to be identical at 20 mg/mL. When assessing the inhibition zone diameter, no inhibition zones were observed at concentrations of 10, 20, and 40 mg/mL of *S. hortensis* EO. Nevertheless, as the concentration of the EO increased to 320 mg/mL, the diameter of the inhibition zone was significantly expanded for all studied bacteria, with inhibition zone diameters at this concentration against *S. aureus* , *S. typhimurium* , *E. coli* , and *L. monocytogenes* were measured as 17.5, 16.26, 24.86, and 22.23 mm, respectively. The results demonstrated that the most sensitive bacterium to this EO was *E. coli* , whereas the most resistant was *S. typhimurium* . In accordance with the present study, Abou Baker et al. (2020) examined the antimicrobial impact of summer *S. hortensis* EO against *E. coli* , *S. typhimurium* , *L. monocytogenes* , and *S. aureus* , reporting the highest inhibitory effect against *E. coli* . The effect of the EO on various microorganisms revealed that *S. hortensis* EO was effective against Gram‐positive and Gram‐negative bacteria, fungi, and yeasts. However, its effectiveness varied depending on the type of organism, where in all microorganisms, the diameter of the inhibition zone grew with higher EO concentrations (Skočibušić et al. 2006). *S. hortensis* EO presented inhibition zones for *E. coli* and *Salmonella* bacteria as 38 and 32 mm, respectively. This strong antibacterial effect may be assigned to the high thymol concentration (41.28%) in the EO (Seyedtaghiya et al. 2021). In a study by Mihajilov‐Krstev et al. (2009), the antibacterial properties of summer *S. hortensis* EO were reported against both Gram‐negative and Gram‐positive bacteria, with MIC values ranging from 0.78 to 25 μL/mL and MBC values between 0.05 and 0.78 μL/mL, well representing significant antimicrobial effects. Previous research showed different MIC and MBC values for *S. hortensis* EO which may be owing to plant distribution area and genetic characteristics of the examined strain (Alizadeh Behbahani et al. 2021; Seyedtaghiya et al. 2021; Sabahi et al. 2022). The inhibitory effect of summer *S. hortensis* EO against bacteria and fungi could be assigned to the elevated content of bioactive compounds from the monoterpene class, particularly terpinene, thymol, and carvacrol (Hamidpour et al. 2014). Further, Farzaneh et al. (2015) reported that the primary active components of summer savory consist of terpenes, penetrating the lipid structure of microbial cell walls and inducing cell death by denaturing proteins as well as causing cytoplasmic leakage.

**TABLE 2:** Antibacterial activity of *S. hortensis* essential oil against food pathogenic organisms by micro broth dilution method.

Strains	MIC (mg/mL)	MBC (mg/mL)
*Staphylococcus aureus*	20	20
*Escherichia coli*	20	20
*Salmonella Typhimurium*	20	20
*Listeria monocytogenes*	20	20

**TABLE 3:** Antibacterial activity of *S. hortensis* essential oil against food pathogenic organisms by disk diffusion method.

*S. hortensis* EO (mg/mL)	*Staphylococcus aureus* (mm)	*Escherichia coli* (mm)	*Salmonella Typhimurium* (mm)	*Listeria monocytogenes* (mm)
10	—	—	—	—
20	—	—	—	—
40	—	—	—	—
80	6.33 ± 0.15^c^	9.00 ± 1.00^c^	9.00 ± 0.20^c^	9.90 ± 0.36^c^
160	11.00 ± 0.50^b^	14.73 ± 0.64^b^	14.43 ± 0.51^b^	13.26 ± 0.87^b^
320	17.50 ± 0.50^a^	24.86 ± 0.80^b^	16.26 ± 0.25^a^	22.23 ± 0.80^b^

*Note:* The mean ± SD within columns with different small letters differs significantly (*p* < 0.05).

### 3.4 Determination of Size and Zeta Potential of *S. hortensis* EO Nanoemulsion

In this study, the diameter of the *S. hortensis* EO nanoemulsion was measured using Dynamic Light Scattering (DLS) testing. The average particle diameter of the *S. hortensis* EO nanoemulsion was measured to be 135.6 ± 3.81 nm. Further, the zeta potential of the particles in this nanoemulsion was found to be −51.26 ± 1.70 mV on average, demonstrating the presence of a negative charge on the particles. Based on previous studies, the size of droplets and stability of nanoemulsions formed through ultrasonication can be influenced by various operational parameters, including power, amplitude, time, and temperature as well as the surfactant type and surfactant to oil ratio (Hashtjin and Abbasi 2015; Mazarei and Rafati 2019). Consistent with the current results, Maccelli et al. (2020) found that the average particle size of *S. hortensis* EO nanoemulsion at four concentrations was approximately 81.3 nm, and as the diluent increased, the particle size diminished. They also reported negative particle potential values, averaging −15.6 mV. Similarly, the particle size of *S. hortensis* EO nanoemulsion was reported to lie within the range of 109 to 118 nm which had fewer changes after 2 weeks. They also found that the zeta potential of the particles in this nanoemulsion presented a high negative charge of approximately −38 mV, confirming its stability (Hashemi et al. 2024).

### 3.5 Microbiological Analysis

#### 3.5.1 TVC

**FIGURE 1:**
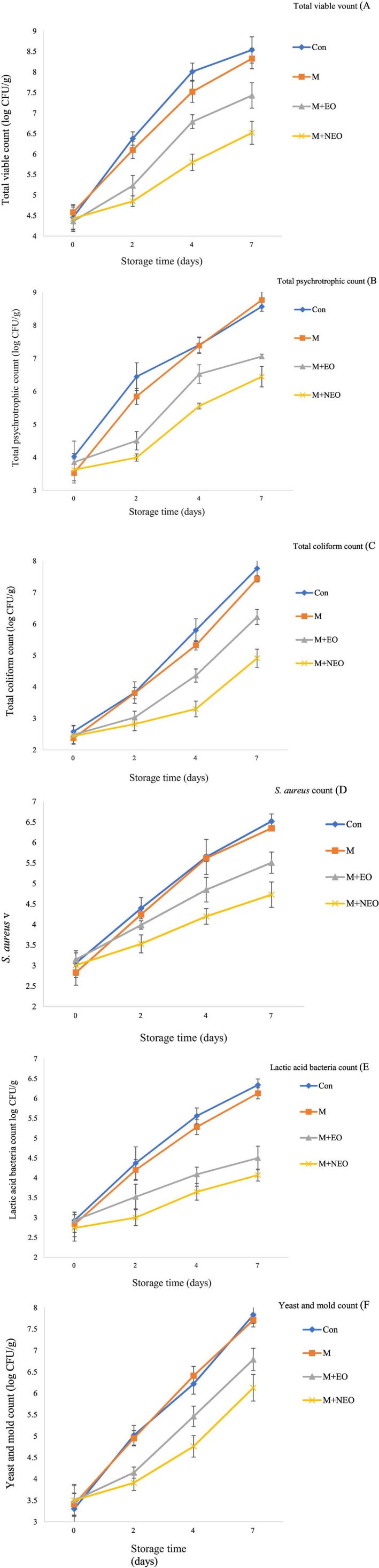
Changes in total viable vount (A) Total psychrotrophic count (B) Total coliform count (C) *S. aureus* count (D) Lactic acid bacteria count (E) Yeast and mold count (F) of rainbow trout fillet stored at 4°C.

Figure 1 displays the microbial variations (including TVC, psychrotroph, coliform, *S. aureus* , LAB, as well as yeast and mold) in rainbow trout fillets packaged with edible coatings containing *D. moldavica* seed mucilage, *S. hortensis* EO, and *S. hortensis* EO nanoemulsion along the study period. The initial microbial load of freshwater fish varies depending on temperature and water conditions. Based on studies, TVC is considered acceptable within the range of 2 to 6 log CFU/g to ensure the freshness of fishery products (Zhaleh et al. 2019). As depicted in Figure 1A, the TVC rose with the number of storage days, aligning with findings from other studies (Sabahi et al. 2022). The TVC in rainbow trout fillets across all four groups examined was in the range of 4.36 to 4.58 log CFU/g on day 0, with no significant differences observed among the groups. By day 7, the total bacterial counts in the CON and M groups were 8.54 log CFU/g and 8.33 log CFU/g, respectively, whereas in the M + EO and M + NEO groups, the total counts reached 7.43 log CFU/g and 6.52 log CFU/g, respectively. In the present study, the nanoemulsion of *S. hortensis* EO was found to inhibit microbial growth more effectively than the EO emulsion did, with the TVC at the end of the storage period reaching 7.43 log CFU/g in the M + EO group and 6.52 log CFU/g in the M + NEO group. In this regard, Sabahi et al. (2022) explored the properties of cinnamon EO and its application in a mucilage coating of *M. sylvestris* seeds, reporting similar findings regarding the extension of shelf life of lamb. They noted that the TVC was significantly elevated in all groups during storage, but in the mucilage group containing 2% cinnamon EO, it did not exceed the permissible limit by day 10 of the study; on the other hand, in the present study, the M + EO group exceeded the acceptable level at day 7. This difference could be attributed to benzyl benzoate (41%) and caryophyllene oxide (26%) in cinnamon EO. In accordance with this study, Zanganeh et al. (2023) reported that the lowest TVC was linked to edible coating containing *Lallemantia iberica* seed mucilage and 2% citrus paradise EO nanoemulsion (6.26 log CFU/g) in lamb samples along storage at 4°C when compared with other concentrations studied. Hashemi et al. (2024) reported that savory‐loaded nanoemulsion revealed greater antibacterial activity against *Enterococcus faecium* in comparison to savory‐loaded emulsions, and they attributed this to the smaller droplet size (109.27 to 118.55 nm), superior surface area, and less surface tension. Shahrampour and Razavi (2023) evaluated the impact of *Eremurus luteus* root gum coating incorporated with rosemary EO nanoemulsions on chicken fillets along cold storage. They reported that the properties of the coating were affected by the concentration of the EO than by their droplet size; the microbial count in fillets coated with 4% nanoemulsion reached (> 12th day) 7 log CFU/g, while in the current study with 2% nanoemulsion, the total count did not reach 7 log CFU/g until the last studied day.

#### 3.5.2 Psychrotrophs

Psychrotrophic bacteria are a group of microorganisms that proliferate at low temperatures, leading to undesirable physicochemical and organoleptic changes in seafood, resulting in reduced shelf life of marine products (Vieira et al. 2019). Based on Figure 1B, the count of psychrotrophic bacteria in the CON group on day 0 was 4.03 log CFU/g, which rose to 8.57 log CFU/g after 7 days. The maximum permissible level of psychrotrophic bacteria in food is 7 log CFU/g, beyond which signs of spoilage begin to appear (Kiakojori et al. 2024). The count of psychrotrophic bacteria in fish samples grew throughout the storage period in all groups, in line with the study by Alizadeh Behbahani and Imani Fooladi (2018). The rate of growth during the study was higher in the control group than in the other groups, with signs of spoilage, including unpleasant odors, noted after 7 days (Kiakojori et al. 2024). No significant differences were observed in the psychrotroph counts on days 4 and 7 between the CON and M groups; however, significant differences were found between these two groups and the M + EO and M + NEO groups, demonstrating the antimicrobial properties of the EO and the gradual release of antimicrobial compounds (Eslamian Amiri et al. 2021). This could be owing to the effects of monoterpene compounds such as carvacrol present in *S. hortensis* EO, penetrating the lipid structure of cell walls of microorganisms and causing cell death by denaturing proteins as well as leaking cytoplasm (Farzaneh et al. 2015). Tanavar et al. (2021) reported that the number of psychrotrophs in veal coated with an edible covering based on basil seed mucilage containing 2% oregano EO rose more slowly compared to the other samples. In line with the results of the present study, Alizadeh Behbahani and Imani Fooladi (2018) concluded that the rate of increase in psychrotrophic bacteria in 9 days of storage for beef coated with Balangu seed mucilage and Feverfew EO was lower compared to other samples. Noshad et al. (2020) stated that mucilage‐based coatings of plant seeds can function as a barrier against oxygen diffusion, as well as inhibit the contact between the sample surfaces and oxygen. As a result, the growth rate of psychrotrophic bacteria in the coated samples diminished compared to the CON. In the present study, the M group could not affect the microbial count of samples. This can be because of the concentration of mucilage (1%) in the current study in comparison to Noshad et al. (2020) which employed 2% mucilage.

#### 3.5.3 Coliform

Based on the results (Figure 1C), the initial count of coliforms in the CON group was 4.03 log CFU/g. The rate of increase in coliforms in the CON group was far higher than in the groups containing *S. hortensis* EO, with the coliform count in the CON group reaching 7.76 log CFU/g by day 7. However, in the strongest groups, namely M + EO and M + NEO, the counts were 6.22 log CFU/g and 4.91 log CFU/g, respectively. Consistent with the current research, Heydari et al. (2020) ascertained the mucilage coating of *Shirazi Qodomeh* seeds containing lavender EO in ostrich meat. They found that the number of coliform bacteria increased along storage in all treatments, but on day 9 of storage, there was a significant difference between the CON sample (7.17 log CFU/g) and samples containing 2% EO (3.34 log CFU/g) (Heydari et al. 2020). Also, in a similar study, Barzegar et al. (2020) explored the effects of an edible coating containing *Heracleum lasiopetalum* EO and *lepidium sativum* seed mucilage over 9 days of beef storage. They stated that the coliform count in groups containing mucilage and 1.5% EO was lower than in other groups.

#### 3.5.4 *S. aureus*

Figure 1D illustrates the changes in the count of *S. aureus* in rainbow trout fillets packaged with *D. moldavica* seed mucilage, *S. hortensis* EO, and *S. hortensis* EO nanoemulsion during refrigeration storage. The count of *S. aureus* in the fish fillet on day 0 was 3.06 log CFU/g, which rose to 6.52 log CFU/g and 6.35 log CFU/g in the CON and M groups after 7 days at refrigeration temperature, respectively. On the contrary, the mucilage group containing the emulsion and nanoemulsion of *S. hortensis* EO revealed a count approximately 1.1–1.5 log lower, representing the antimicrobial properties of *S. hortensis* EO. Likewise, Tanavar et al. (2021) reported that the control group and groups coated with *O. basilicum* seed mucilage and *M. pulegium* EO presented the largest and the smallest changes regarding *S. aureus* count in veal. *S. aureus* is a Gram‐positive, facultative anaerobic bacterium. Thus, this bacterium grew better in the control sample, as it was not coated. Further, the role of phenolic compounds and the antioxidant effect of EOs have been noted in the literature (Barzegar et al. 2020). In a similar study inspecting the effects of Balangu seed mucilage and 2% Feverfew EO edible coating on beef samples, the increase in *S. aureus* count was less than in other samples (Alizadeh Behbahani and Imani Fooladi 2018).

#### 3.5.5 LAB

The findings of the changes in the total count of LAB in rainbow trout fillets packaged with *D. moldavica* seed mucilage, *S. hortensis* EO, and *S. hortensis* EO nanoemulsion along refrigeration presented a similar trend to the TVC (Figure 1E). Throughout the study, no significant changes were observed between the CON group and M group. The lowest count of LAB at the end of the storage period was related to the groups containing EO and nanoemulsion; however, a significant difference was found between the M + EO and M + NEO groups, with the nanoemulsion group revealing a lower count of LAB. In line with our result, Pirastehfard et al. (2021) reported the inhibitory effect of canola oil nanoemulsion combined with *Satureja Bachtiarica* EO on LAB in chicken breast. Elsewhere, Agdar GhareAghaji et al. (2021) examined the effects of salep containing orange EO on rainbow trout fillets. Similar to the findings of the current study, the initial LAB count was about 3.08 Log CFU/g, representing the freshness of the fish. During storage, the number of LAB rose in all treatments, with the highest number in control. Ghaderi‐Ghahfarokhi et al. (2017) reported that preparing beef patties with 0.1% encapsulated cinnamon EO incorporated chitosan nanoparticles could keep LAB count below 4 log CFU/g up to the 8th day, whereas in the present study, the LAB count reached 4.07 log CFU/g on day 7. This discrepancy could be attributed to the fact that in the current study the fillets were coated with the edible coating which needs the coating to be absorbed by the food to affect.

#### 3.5.6 Yeast and Mold

No significant differences were observed between CON and M groups regarding yeast and mold count over the study period (Figure 1F). The highest growth inhibition of yeast and mold was observed in the groups containing the emulsion and nanoemulsion of *S. hortensis* EO, with the strongest group, M + NEO, presenting a yeast and mold count of 6.13 log CFU/g on day 7. In accordance with the present study, researchers found that the edible coating of *Lepidium perfoliatum* seed mucilage containing chicory EO inhibited the growth of fungi in beef samples (Alizadeh Behbahani et al. 2021). The positive effect of edible coatings containing mucilage from plant seeds combined with EOs could be mainly assigned to the antimicrobial activity of the EO (Behbahani and Imani Fooladi 2018). Similarly, a biologically active edible coating of sage seed mucilage and *Myristica fragrans* EO significantly reduced the population of fungi in comparison to control significantly (Kiarsi et al. 2020).

### 3.6 Chemical Analysis

#### 3.6.1 pH

pH is an important indicator of fish freshness, where changes in pH can serve as a spoilage index in fish. The findings (Table 4) indicated that the pH on day 0 was 6.28 and rose by 0.59 during the study period, while the pH in M + NEO increased by 0.36. The pH of fresh fish ranges from 6.1 to 6.3, but it grows over time (Brewer et al. 2006). An increase in pH can indicate bacterial growth, reduced quality, protein degradation, microbial metabolites, and ultimately spoilage of fish (Kiliç et al. 2014; Hassanzadeh et al. 2018). A similar reduction in pH has been observed with active and edible coating on sardine fillets (Homayonpour et al. 2021). In accordance with the current study, Noshad et al. (2020) found that the pH of shrimp in the groups coated with quince seed mucilage containing green tea extract was lower than that of the control shrimp, attributed to the effectiveness of coating in postponing spoilage and decomposition of the shrimp. The positive effect of mucilage coating containing EOs has been attributed to the reduction of microorganisms growth, slow degradation of food texture, decline of carbon dioxide permeability, and accumulation of carbon dioxide generated by meat respiration (Behbahani et al. 2017).

**TABLE 4:** Changes in pH (A), TVB‐N (B), and TBARS (C) of rainbow trout fillet stored at 4°C.

	Treatment	Storage time (days)			
		0	2	4	7
pH	Con	6.28 ± 0.03^aD^	6.37 ± 0.01^aC^	6.58 ± 0.02^aB^	6.87 ± 0.01^aA^
	M	6.29 ± 0.01^aC^	6.32 ± 0.01^bC^	6.52 ± 0.02^bB^	6.81 ± 0.01^bA^
	M + EO	6.29 ± 0.02^aC^	6.29 ± 0.00^cC^	6.39 ± 0.01^cB^	6.70 ± 0.02^cA^
	M + NEO	6.28 ± 0.01^aC^	6.26 ± 0.01^dC^	6.35 ± 0.03^dB^	6.64 ± 0.02^dA^
TVB‐N	Con	11.46 ± 0.44^aD^	21.05 ± 0.52^aC^	28.51 ± 0.46^aB^	39.52 ± 0.22^aA^
	M	11.21 ± 0.22^aD^	20.88 ± 0.41^aC^	27.63 ± 0.22^bB^	39.12 ± 0.32^aA^
	M + EO	11.55 ± 0.34^aD^	18.53 ± 0.38^bC^	23.91 ± 0.38^cB^	31.61 ± 0.41^bA^
	M + NEO	8.58 ± 0.37^aA^	7.58 ± 0.23^aB^	7.18 ± 0.20^aBC^	6.86 ± 0.15^bC^
TBARS	Con	0.12 ± 0.02^aD^	0.46 ± 0.01^aC^	0.72 ± 0.03^aB^	0.89 ± 0.03^aA^
	M	0.11 ± 0.03^aD^	0.42 ± 0.02^abC^	0.63 ± 0.02^bB^	0.76 ± 0.02^bA^
	M + EO	0.12 ± 0.02^aB^	0.24 ± 0.18^bB^	0.46 ± 0.02^cA^	0.62 ± 0.03^cA^
	M + NEO	0.12 ± 0.02^aD^	0.24 ± 0.04^bC^	0.38 ± 0.02^dB^	0.51 ± 0.02^dA^

*Note:* Values represent mean ± standard deviation (*n* = 3). Means in the same row with different capital letters are significantly different (*p* < 0.05). Means in the same column with different small letters are significantly different (*p* < 0.05). Uncoated (CON), 1% *D. moldavica* mucilage coating (M), mucilage coating containing 2% *S. hortensis* EO (M + EO), and mucilage coating containing 2% nanoemulsion of *S. hortensis* EO (M + NEO).

#### 3.6.2 TVB‐N

Table 4 reports the results of measuring TVB‐N in rainbow trout fillets packaged with edible coatings containing *D. moldavica* seed mucilage, *S. hortensis* EO, and *S. hortensis* EO nanoemulsion along the storage period. The type and amount of TVB‐N serve as the most important and common quality indicator of fish spoilage, comprising amine compounds (trimethylamine, dimethylamine, ammonia, and other similar compounds) generated by the activity of endogenous enzymes and microbial action (Socaciu et al. 2018). As stated by Morachis‐Valdez et al. (2017), the maximum acceptable level of TVB‐N in fish tissue ranges from 25 to 40 mg/100 g. In this study, the levels of volatile nitrogenous compounds revealed no significant differences among the groups on day 0. Throughout the study, the TVB‐N levels rose owing to the deterioration of muscle tissue stability caused by spoilage bacteria. On day 7, the highest TVB‐N level was observed in the CON group (39.52 mg/100 g), whereas the lowest was in the M + NEO group (28.59 mg/100 g). In this regard, Noshad et al. (2020) found that quince seed mucilage coating with green tea extract could help mitigate the ascending trend of TVB‐N in shrimp along the study period. Our results are also in line with those of Zomorodian et al. (2023) who stated that the lowest TVB‐N value was linked to salmon coated with chitosan incorporated with *Zataria multiflora* EO Pickering emulsion, while the highest one was associated with the control and chitosan‐coated salmon.

#### 3.6.3 TBARS

TBARS is an indicator employed to evaluate the degree of lipid oxidation in seafood and other meat products (Alparslan et al. 2014), which can result in changes in taste, color, and odor, while also contributing to texture deterioration in fish products (Wenjiao et al. 2013). The TBARS levels in the groups with mucilage containing *S. hortensis* EO and nanoemulsion presented an upward trend, but the rate of growth was significantly lower than that of the CON group, with TBARS levels on day 7 reaching 0.62 and 0.51 mg MDA/kg in the M + EO and M + NEO groups, respectively (Table 4). The initial TBARS level in the present study was 0.12 mg MDA/kg, which is in line with results of Agdar GhareAghaji et al. (2021) who reported the initial TBARS in rainbow trout fillets to be approximately 0.15 mg MDA/kg. According to the results, a significant difference was observed between CON and M groups after the 4th day. This may be attributed to the barrier properties of the edible coatings and its potential to lower lipid oxidation (Coban and Coban 2020). In a similar study, the minimum and maximum TBARS values of refrigerated beef were samples coated with whey protein coating containing nanoemulsion of *Trachyspermum copticum* EO and control, respectively. This may be owing to the slow release of EO on the surface of food (Saghari et al. 2024).

### 3.7 Sensory Analysis

The organoleptic properties (taste, color, odor, texture, and overall acceptability) of rainbow trout fillets packaged with edible coatings containing *D. moldavica* seed mucilage, *S. hortensis* EO, and *S. hortensis* EO nanoemulsion over the study period are outlined in Table 5. Based on the results, the trend of changes in the factors of taste, texture, odor, and overall acceptability diminished with the prolongation of the storage time in all groups. Nevertheless, the highest scores were related to the M + EO and M + NEO groups, representing the favorable impact of *S. hortensis* EO and its nanoemulsion on the organoleptic properties of the fish fillets. Beef slices coated with *Lepidium perfoliatum* seed mucilage containing chicory EO presented the highest oxidative and microbial stability and received the highest sensory scores (Alizadeh Behbahani et al. 2021). It has been proven that elevating the concentration of rosemary EO nanoemulsions in the *Eremurus luteus* root gum coating improved the sensory parameters of the chicken fillet (Shahrampour and Razavi 2023). In accordance with the results of the current study, *M. sylvestris* mucilage coating enriched with 2% *Cinnamomum zeylani* EO could preserve the sensory parameters of lamb meat slices along cold storage (Sabahi et al. 2022). The oxygen/water barrier properties and presence of antimicrobial as well as antioxidant compounds in edible coatings containing plant EOs would delay microbial and chemical spoilage of protein products, including meat, thereby preserving and improving the organoleptic properties of these products in comparison to the control (Alizadeh Behbahani et al. 2021; Sabahi et al. 2022).

**TABLE 5:** Changes in sensory scores of rainbow trout fillet stored at 4°C.

Sensory attributes	Treatment	Storage time (days)			
		0	2	4	7
Taste	Con	8.53 ± 0.45^aA^	8.36 ± 0.58^aA^	6.62 ± 0.23^bB^	—
	M	8.23 ± 0.25^aA^	8.06 ± 0.47^abA^	6.77 ± 0.25^bB^	—
	M + EO	8.13 ± 0.41^aA^	7.56 ± 0.12^bAB^	7.35 ± 0.29^aB^	—
	M + NEO	8.15 ± 0.27^aA^	7.65 ± 0.19^abB^	7.48 ± 0.16^aB^	—
Color	Con	8.75 ± 0.31^aA^	7.80 ± 0.26^aB^	6.19 ± 0.19^bC^	4.21 ± 0.30^cD^
	M	8.65 ± 0.30^aA^	7.53 ± 0.27^aB^	6.20 ± 0.26^bC^	4.47 ± 0.41^aD^
	M + EO	8.56 ± 0.40^aA^	7.45 ± 0.11^aB^	7.00 ± 0.11^aC^	6.33 ± 0.17^bD^
	M + NEO	8.58 ± 0.37^aA^	7.58 ± 0.23^aB^	7.18 ± 0.20^aBC^	6.86 ± 0.15^bC^
Odor	Con	8.76 ± 0.25^aA^	7.90 ± 0.36^aB^	6.26 ± 0.58^bC^	3.53 ± 0.35^dD^
	M	8.59 ± 0.44^aA^	7.60 ± 0.49^aB^	6.63 ± 0.40^bC^	4.19 ± 0.30^cD^
	M + EO	8.33 ± 0.17^aA^	8.11 ± 0.11^aAB^	7.81 ± 0.17^aB^	6.89 ± 0.28^bC^
	M + NEO	8.39 ± 0.14^aA^	8.16 ± 0.20^aAB^	7.88 ± 0.31^aBC^	7.60 ± 0.22^aC^
Texture	Con	8.86 ± 0.12^aA^	8.25 ± 0.21^aB^	6.20 ± 0.30^bC^	3.40 ± 0.42^cD^
	M	8.73 ± 0.25^aA^	8.20 ± 0.22^aB^	6.71 ± 0.20^bC^	4.02 ± 0.24^bD^
	M + EO	8.73 ± 0.25^aA^	8.32 ± 0.15^aB^	8.00 ± 0.11^aB^	7.21 ± 0.25^aC^
	M + NEO	8.82 ± 0.11^aA^	8.26 ± 0.08^aB^	8.02 ± 0.48^aBC^	7.69 ± 0.14^aC^
Overall acceptability	Con	8.76 ± 0.25^aA^	8.20 ± 0.26^aB^	6.56 ± 0.20^bC^	4.38 ± 0.34^cD^
	M	8.78 ± 0.18^aA^	8.05 ± 0.19^aB^	6.93 ± 0.22^bC^	4.81 ± 0.27^cD^
	M + EO	8.54 ± 0.44^aA^	8.07 ± 0.49^aAB^	7.74 ± 0.22^aB^	6.51 ± 0.37^bC^
	M + NEO	8.59 ± 0.43^aA^	8.10 ± 0.06^aB^	7.99 ± 0.13^aB^	7.11 ± 0.18^aC^

*Note:* Values represent mean ± standard deviation (*n* = 3). Means in the same row with different capital letters are significantly different (*p* < 0.05). Means in the same column with different small letters are significantly different (*p* < 0.05). Uncoated (CON), 1% *D. moldavica* mucilage coating (M), mucilage coating containing 2% *S. hortensis* EO (M + EO), and mucilage coating containing 2% nanoemulsion of *S. hortensis* EO (M + NEO).

## 4 Conclusion

Overall, the edible coating containing *D. moldavica* mucilage and *S. hortensis* EO emulsion as well as nanoemulsion could improve microbial, chemical, and sensory properties of rainbow trout fillets, leading to an extension of shelf life by more than 3 days. Thus, *D. moldavica* mucilage containing emulsion and nanoemulsion of *S. hortensis* EO could be employed as a biodegradable coating to boost the shelf life of food products. The limitation of the present study was the use of only one concentration of the EO in the form of emulsion and nanoemulsion in the edible coating. It is suggested that researchers utilize a range of EO concentrations to compare their effectiveness as well as use other plant mucilage along with *S. hortensis* EO to create edible coatings.

## Author Contributions

**Kosar Maghsoudi:** funding acquisition (equal), investigation (lead), methodology (lead), resources (equal), writing – original draft (lead). **Razieh Partovi:** conceptualization (lead), funding acquisition (equal), project administration (lead), resources (equal), supervision (lead), writing – review and editing (lead). **Shohreh Alian Samakkhah:** data curation (lead), formal analysis (lead), validation (lead), visualization (lead).

## Conflicts of Interest

The authors declare no conflicts of interest.

## Data Availability

The datasets generated and analyzed during the current study are available from the corresponding author on a reasonable request.
